# Association between the 400-m walk test and sensor-based daily physical activity in frail and sarcopenic older adults

**DOI:** 10.1007/s41999-025-01262-4

**Published:** 2025-06-26

**Authors:** Jana Rogler, Sebastian Krumpoch, Ellen Freiberger, Ulrich Lindemann, Robert Kob

**Affiliations:** 1 Department of Internal Medicine-Geriatrics, Institute for Biomedicine of Ageing (IBA), Friedrich-Alexander-Universität Erlangen-Nürnberg, Nuremberg, Germany; 2 Department of Geriatrics, Robert Bosch Hospital, Stuttgart, Germany

**Keywords:** 400-m walk test, Daily activity, Older adults, Frailty, Sarcopenia

## Abstract

**Purpose:**

Physical activity (PA) is recommended for frail and sarcopenic older adults as an essential means of preventing negative health outcomes and decline in functional abilities. Our objective was to examine the association between the time and/or stops during the 400-m walk test (400MWT) and average daily steps and walking cadence in a German cohort of frail and sarcopenic community-dwelling older adults.

**Methods:**

For this sub-study the German cohort of 104 frail and sarcopenic older adults (i.e., SPRINTT) aged 80.8 ± 5.2 years was divided into participants having made none or one stop or more than one stops, referred to as non-stoppers and multi-stoppers. The characteristics and general health state (physical function, disease state, concerns about falling) at first observation (FO) and individual last observation (LO) after at least 11 months (mean 24.5 ± 8.5 months) were examined. Daily PA represented by average daily steps and walking cadence was assessed over three to seven days at FO and LO using the activPAL3 micro. Time and stops made during the 400MWT and their association with daily PA were investigated using regression with bootstrapping.

**Results:**

Out of 104 frail and sarcopenic older adults, 84 non-stoppers (female: *n* = 54; 64.3%) had a median time in 400MWT of 509 s (Inter Quartile Range (IQR) 324–875), a median number of daily steps of 6537 (IQR 1841–19,488) and a median daily walking cadence of 73 steps/minute (IQR 53.3–88.9). 20 multi-stoppers (female: *n* = 12; 60%) showed a time of 703 s (IQR 479–898), 5642 steps (IQR 2470–11,458) and a cadence of 70.7 steps/minute (IQR 61.4–83.6). Time was significantly associated with average daily steps and walking cadence at both FO and LO, stops alone were not.

**Conclusion:**

Gait speed under laboratory conditions can be used in clinical settings and research to estimate daily PA, represented by average daily steps and cadence, in frail and sarcopenic older adults.

## Key summary points

**Aim:**

Our objective was to examine the association between the time and number of stops during the 400-meter walk test (400MWT) and average daily steps and walking cadence in a German cohort of frail and sarcopenic community-dwelling older adults at first observation (FO) and individual last observation (LO) after at least 11 months.

**Findings:**

Stops during the 400MWT led to a longer time. Time in the 400MWT was significantly associated with average daily steps and walking cadence at FO and LO (24.5 ± 8.5 months). Stops alone were not associated with average daily steps and walking cadence.

**Message:**

Gait speed under laboratory conditions can be used to estimate daily physical activity, represented by average daily steps and walking cadence, in frail and sarcopenic older adults.

## Introduction

Physical activity (PA) is recommended for older adults as an essential means of preventing negative health outcomes and decline in functional abilities [1, 2]. Increased muscle weakness due to age-associated muscle loss, physical inactivity, diseases, and malnutrition lead to a reduction in daily mobility and ultimately to the development of sarcopenia [3, 4]. This muscle disease contributes to the pre-disability geriatric condition of frailty [5], defined as increased risk for mortality and immobility when exposed to a stressor [6, 7]. These age-related conditions make it difficult for those affected to comply with prevalent activity recommendations [3, 8]. Moreover, men and women show different activity patterns [9, 10] and approaches to influencing them must be consciously chosen. It is key for health professionals to determine the mobility status of frail and sarcopenic older adults [8] to provide them with adapted activity recommendations for their daily routine, since both frailty and sarcopenia can be positively influenced by mobility interventions [2, 11, 12]. Unfortunately, the assessment of PA in the in-patient setting is not representative for daily PA in the home setting. However, the use of wearable technologies, which accurately measure PA like gait, in the form of steps and cadence, is limited by cost and accessibility for many older adults.

The 400MWT is a widely used and validated tool to assess mobility disability in geriatric research [13, 14]. Rolland et al. (2004) prioritized the ability to complete the test within 15 min at usual gait speed to objectively examine mobility disability. The 400MWT provides information about the time and the number of stops made during the test [13]. But so far, only few studies have focused on the number of stops in walking tests as additional sign of mobility disability. Research in patients with chronic obstructive pulmonary disease (COPD) or peripheral arterial disease (PAD) who underwent the 6-min walk test (6MWT) showed an association between a higher number of stops and less distance walked [15, 16] and even a higher risk for long-term mortality and hospitalization [17]. To the best of the authors' knowledge, only one study took a closer look at the stops in the 400MWT and concluded a strong association between stopping and mobility disability in nondisabled community-dwelling older adults with functional limitations, which may be explained by the participants’ poor physical functioning [18]. Another explanation may be an individual’s walking strategy, which can manifest itself in continuous versus intermittent walking [19] or high versus low walking cadence [20], and may also have an impact on the number of stops and activity behavior. These factors are in turn influenced by age-related physiological changes, that can lead to changes in gait parameters such as a decrease of daily steps and walking cadence [21, 22].

It has been demonstrated that the development of age-related functional impairment and consequently mobility disability can be reduced by changes in frequency and amount of PA [23], also shown in the SPRINTT study [24], part of whose data is analyzed in the sub-study described below.

This raises the question of whether time and/or number of stops made in the 400MWT could indicate daily PA in frail and sarcopenic older adults. To the best of the authors’ knowledge, this association has not yet been investigated. This information may be a future basis to provide more individualized recommendations for PA. We expect older adults with faster test times to have made less stops in the 400MWT. Furthermore, we hypothesize that time and number of stops are significantly associated with average daily steps and walking cadence (steps per minute), assessed over a wearing time of 3 to 7 days at first observation (FO) and individual last observation (LO) performed after 11 up to 36 months using the activPAL3 micro. Due to age-related functional decline, we expect a decrease in average daily steps and walking cadence over the period of FO and individual LO. Additionally, we assume that older adults with faster times and fewer stops in the 400MWT show a significantly higher daily average of step number and walking cadence, regardless of sex, age and group.

## Methods

### Study design

This sub-study is a supplementary analysis of the SPRINTT trial that was conducted between January 2016 and October 2019 as a single-blinded, randomized multi-centric project (ClinicalTrials.gov identifier: NCT02582138). It was performed at 16 study centers across eleven European countries. The study protocol was approved by the ethics committee of the Università Cattolica del Sacro Cuore, Rome, Italy (protocol No 15611/15), and was subsequently ratified by the ethics committees of all participating institutions. All participants signed an informed consent form. In depths, information on the SPRINTT study, including protocol, has been published [11, 24].

The aim of the SPRINTT study was to compare the effectiveness of a multicomponent intervention (MCI) with a healthy aging lifestyle education (HALE) program for preventing mobility disability in initially non-disabled older adults with physical frailty and sarcopenia [11]. More detailed information can be found in the SPRINTT study protocol [11].

### Participants

**Fig. 1:**
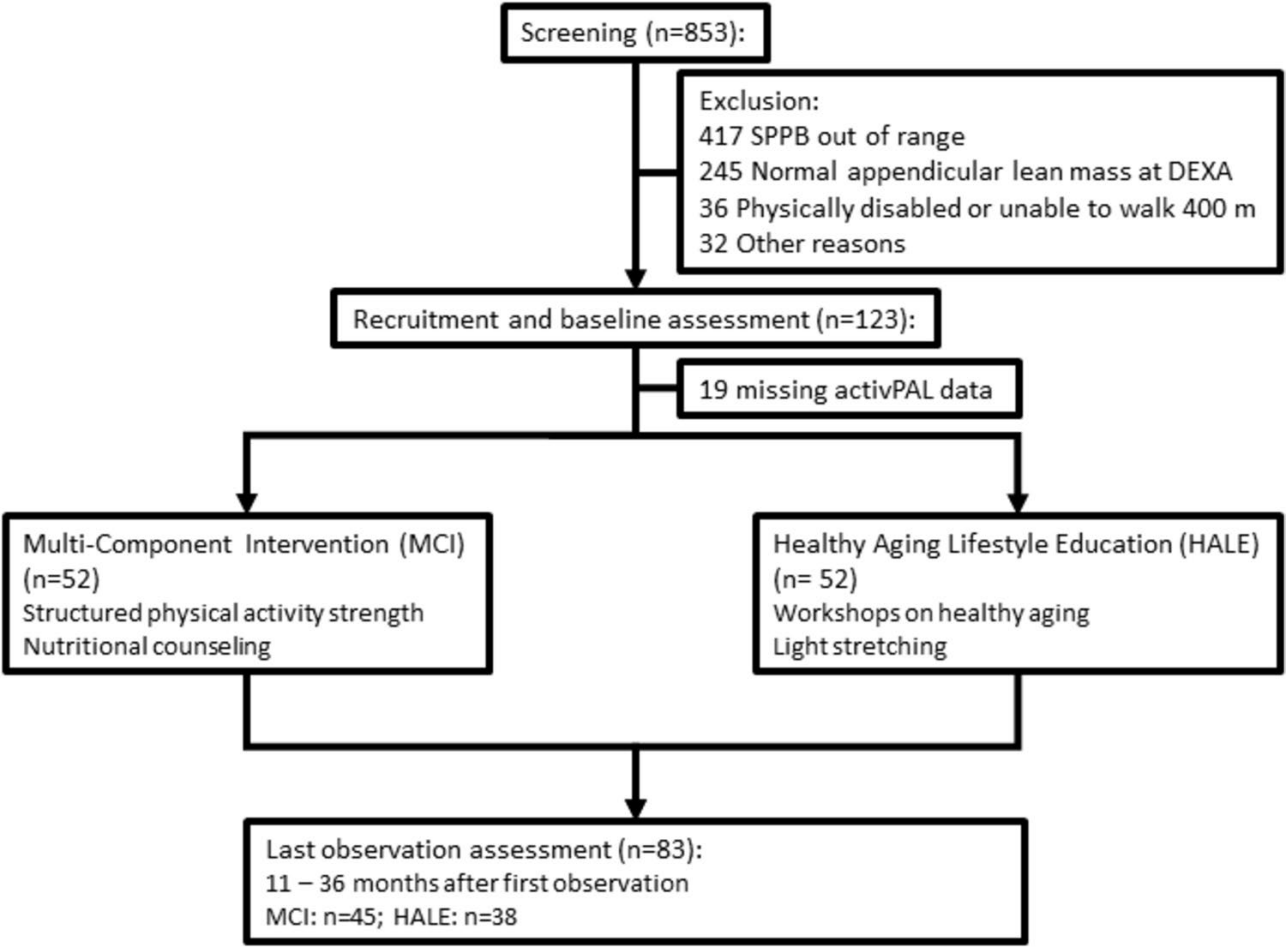
Flow chart of participants through the course of the sub-study

Briefly, the participants were recruited according to the same criteria as in the SPRINTT study (described in detail in [11, 24]). In this sub-study, older adults aged 70 years or older living in the Nuremberg metropolitan area were included (German cohort) (Fig. 1). The inclusion of frail and sarcopenic older adults was based on the Short Physical Performance Battery (SPPB) score of ≥ 3 to ≤ 9 points (score ranges from 0 to 12 points with lower scores indicating poorer physical functioning) [25]. Another inclusion criterion was low appendicular lean mass (aLM) with gender-specific cut-points according to the associations of the Foundation for the National Institutes of Health Sarcopenia Project (FNIH) (< 19.75 kg men and < 15.02 kg women) and low aLM-to-Body-Mass-Index(BMI)-ratio (< 0.789 men and < 0.512 women) [26] that was measured with a dual-energy X-ray absorptiometry (DXA) device. The non-existence of mobility disability was assessed as completing the 400MWT within 15 min without stopping for more than 1 min at a time, sitting or receiving help [13]. In the sub-study, people having made ≤ 1 stop and > 1 stop will be referred to as non-stoppers and multi-stoppers.

Older adults were excluded if they self-reported walking disability, scored ≤ 24 points in Mini-mental state examination (MMSE) [27], have been diagnosed with terminal illness or contraindications to safely engage in study as judged by local study doctors, participated in a structured PA program or moved away from the study area within at least two years. The sub-study also excluded people who wore the activPAL for less than three days. Data were collected every 6 months , while this analysis focused on participants that performed the FO and the individual LO.

### Intervention period

The interventions were administered for up to 36 months, depending on when the participants were recruited.

The *MCI* group practiced moderate physical training and received nutritional counseling. The *PA* was structured the same way as in the LIFE study and included aerobic, strength, flexibility, and balance exercises. The nutritional counseling aimed to supplement the benefits of the PA program. The LIFE protocol has been shown to be safe and effective at preventing mobility disability [28].

The *HALE* group received a *health educational program* through seminars and workshops. Adapted from the LIFE study, it added 5 to 10 min upper extremity stretching or relaxation techniques performed at the end of each session [28]. The HALE group was implemented to strengthen the sense of belonging to this study arm and increased the participants’ perceived benefit without having a direct influence on the study results.

### Outcome measures

#### 400-m walk test

The performance in the 400MWT provides information about mobility disability as the primary outcome. The validity (e.g., objectively measured steps per day *r* = -0.42, *p* < 0.001) and reliability (ICC = 0.95, 95%-CI[0.92, 0.97]) of the 400MWT has been shown in previous studies [14, 29].

Participants were asked to walk a distance of 400 m at their usual pace and without overexerting themselves. During testing, no walking aid other than a single straight cane was allowed. Participants walked laps around two cones placed 20 m apart. Ten laps of 40 m were required to successfully complete the 400MWT. The participants were allowed to stop as often as they wished when exhausted, but for no longer than 60 s at a time. For safety observation, the examiner accompanied the participant along the walking course, staying just behind to avoid influencing the participant’s walking speed. Older adults who could not complete the test at FO within 15 min or had to stop for longer than 60 s were not included. In addition to the time in the 400MWT, the number of the stops made was recorded [13].

### Daily PA

Daily PA data were obtained in both intervention groups using the activPAL3 micro (PAL Technologies Ltd., Glasgow, UK) affixed to the frontal mid-thigh. It has proven to be a valid and reliable tool in measuring sitting, lying, and standing time as well as step number and cadence in community-dwelling older adults [30]. The aim was for the activPAL to be worn for 7 consecutive days at FO and LO within 2 weeks from the scheduled clinical visits to monitor everyday PA represented by daily average steps and walking cadence. In the sub-study, the minimum wearing time was 3 days, as it has been found that a sufficient estimate of weekly PA can be achieved for any three-day combination [31].

### Descriptive measures/covariates

Demographic and anthropometric data such as sex, age, body height and weight were collected. BMI was then computed. Further, handgrip strength was assessed using the Jamar Hand Dynamometer (Sammons Preston, Inc, Warrenville, IL, USA) as a sign of possible functional restrictions and sarcopenia [3]. Physical function was determined using the SPPB [25], whereby only older adults with scores between ≥ 3 and ≤ 9 were included. Concerns about falling were investigated using the Falls Efficacy Scale—International Version (FES-I) [32]. Physical, cognitive and medical condition were obtained by Cumulative Illness Rating Scale (CIRS) [33].

### Data analysis

Data were obtained at two measurement points: first observation (FO) and individual last observation (LO) after at least 11 months.

To characterize the study sample, means and standard deviations for both continuous and discrete variables were used. Categorical variables were presented as percentages.

An unpaired t-test was applied to determine possible group differences between non-stoppers (≤ 1 stop) and multi-stoppers (> 1 stop) with regard to time in 400MWT, average daily steps and walking cadence.

The association between daily activity and stops was examined using logistic regression to investigate the influence of time in 400MWT, average number of daily steps, and walking cadence on stops in 400MWT. Logistic regression was performed using 1.000 bootstrap samples to test the stability of the estimates.

In addition to the stops, we hypothesized that time in 400MWT could indicate PA in everyday life. Multiple linear regression was applied to investigate the relation between time and average number of daily steps and walking cadence, differentiating by sex, age and group (the latter for LO). To investigate the influence on the average number of daily steps at FO**,** stepwise multiple regression with bootstrapping was applied to avoid overfitting to work with a simple and robust model.

A significance level of *α* = 0.05 was used for the entire analysis. The calculations were carried out using SPSS® version 30.0.0.0 (Armonk, NY: IBM Corp).

## Results

Data of 104 participants (mean age at FO: 80.8 ± 5.2 years) were included in the analysis. Average participation time from FO to LO was 24.5 ± 8.5 months. Average performance in 400MWT at FO was 546 ± 136 s and average stops were 1.1 ± 2.1. On average, the participants walked 6362 ± 2927 steps per day with a cadence of 72.5 ± 7.7. A further description of the sample at FO and LO is shown in Table 1, including significant changes over time.

**Table 1:** Description of all older adults included in this sub-study, differentiated by time point

	Mean ± SD (range)	Mean ± SD (range)
Characteristics	FO (*n* = 104)	LO (*n* = 83)
Age [years]	80.8 ± 5.2 (71.0–91.0)**	82.8 ± 5.2 (72.0–93.0)**
Participation time [months]	24.5 ± 8.5 (11.0–36.0)	24.5 ± 8.5 (11.0–36.0)
MCI (%)	52 (50.0)	45 (54.2)
Body height [cm]	159.2 ± 10.3 (141.2–184.5)	159.2 ± 10.3 (141.2–184.5)
Body weight [kg]	75.6 ± 19.8 (44.3–140.0)*	74.7 ± 20.1 (44.5–144.6)*
Body mass index [kg/m^2^]	29.5 ± 6.0 (18.9–49.5)**	29.0 ± 5.8 (18.7–46.7)**
Handgrip strength [kg]	22.1 ± 9.7 (2.0–45.0)**	32.5 ± 9.4 (10.0–52.0)**
SPPB (0–12)	6.8 ± 1.3 (4.0–9.0)**	8.5 ± 2.4 (0.0–12.0)**
CIRS (0–56)	7.2 ± 4.3 (1.0–21.0)**	8.7 ± 3.8 (2.0–19.0)**
FES-I (16–64)	27.7 ± 8.1 (16.0–47.0)	27.0 ± 7.8 (16.0–49.0)
Time 400MWT [s]	546 ± 136 (324–898)	523 ± 135 (288–879)
Stops 400MWT [number]	1.1 ± 2.1 (0.0–14.0)	0.9 ± 2.3 (0.0–13.0)
Average daily steps [number]	6362 ± 2927 (1841–19488)*	5764 ± 3222 (1341–16643)*
Average daily cadence [steps/min]	72.5 ± 7.7 (53.3–88.9)*	70.6 ± 8.1 (49.7–89.2)*

*SD* standard deviation; *SPPB* Short Physical Performance Battery; *CIRS* Cumulative Illness Rating Scale; *FES-I* Falls Efficacy Scale – International Version; *400MWT* 400-m walk test

Statistically significant difference; *p* < 0.05*, *p* < 0.001**

19.3% of all participants stopped more than once during the 400MWT and a connection of time and stops in the 400MWT was evident in the group difference between non-stoppers and multi-stoppers. At FO (Table 2), non-stoppers needed statistically significant less time than multi-stoppers (193 s, 95%-CI[-249, -138], *t*(102) = -6.88, *p* < 0.001). While physical function (measured by SPPB), severity of comorbidities (measured by CIRS), as well as everyday mobility were comparable between groups, the multi-stoppers exhibited higher concerns about falling than non-stoppers (-4.6, 95%-CI[-8.7, -0.5], *t*(99) = -2.23, *p* = 0.027).

**Table 2:** Characteristics of non-stoppers and multi-stoppers (*n* = 104) at FO

	Mean ± SD (range)	
Characteristic	Non-stoppers (*n* = 84; 80.7%)	Multi-stoppers (*n* = 20; 19.3%)
Female (%)	54 (64.3)	12 (60.0)
MCI (%)	42 (50.0)	10 (50.0)
Age	80.9 ± 5.3 (71.0–91.0)	80.4 ± 4.6 (73.0–89.0)
SPPB (0–12)	6.9 ± 1.2 (4.0–9.0)	6.5 ± 1.3 (4.0–9.0)
CIRS (0–56)	6.8 ± 3.9 (1.0–21.0)	8.7 ± 5.6 (1.0–20.0)
FES-I (16–64)	26.8 ± 8.0 (16.0–47.0)*	31.4 ± 7.4 (20.0–44.0)*
Time 400MWT [s]	509 ± 115 (324–875)**	703 ± 105 (479–898)**
Stops 400MWT [number]	0.3 ± 0.4 (0.0–1.0)**	4.6 ± 2.8 (2.0–14.0)**
Average daily steps [number]	6537 ± 2951 (1841–19488)	5642 ± 2785 (2470–11458)
Average daily cadence [steps/min]	73.0 ± 7.9 (53.3–88.9)	70.7 ± 6.5 (61.4–83.6)

*SD* standard deviation; *SPPB* Short Physical Performance Battery; *CIRS* Cumulative Illness Rating Scale; *FES-I* Falls Efficacy Scale – International Version; *400MWT* 400-m walk test

Statistically significant difference; *p* < 0.05*, *p* < 0.001**

The number of stops made during 400MWT did not differ significantly between FO and LO (asymptotic Wilcoxon test: *z* = -0.632, *p* = 0.527, *n* = 84) (Table 1).

18.1% of participants that performed the 400MWT at LO, stopped more than once during the test. Non-stoppers needed statistically less time (208 s, 95%-CI[-270, -146], *t*(81) = -6.70, *p* < 0.001) than multi-stoppers. Additionally, non-stoppers made on average significantly more daily steps (2028 steps, 95%-CI[214.7, 3840.3], *t*(79) = 2.23, *p* = 0.029) than multi-stoppers. While physical function (measured by SPPB), was comparable between groups, the multi-stoppers exhibited higher severity of comorbidities (measured by CIRS) (-2.2, 95%-CI[-4.15, -0.16], *p* = 0.034) (Table 3).

**Table 3:** Characteristics of non-stoppers and multi-stoppers (*n* = 83) at LO

	Mean ± SD (range)			
Characteristic	Non-stoppers (*n* = 68; 81.9%)			Multi-stoppers (*n* = 15; 18.1%)
Female (%)		42 (61.8)	10 (66.7)	
MCI (%)		36 (52.9)	9 (60.0)	
Age		82.5 ± 5.2 (72.0–93.0)	84.2 ± 4.9 (76.0–91.0)	
SPPB (0–12)		8.9 ± 2.2 (4.0–12.0)	7.9 ± 2.1 (4.0–12.0)	
CIRS (0–56)		8.1 ± 3.4 (2.0–18.0)*	10.2 ± 3.4 (5.0–18.0)*	
FES-I (16–64)		25.3 ± 6.4 (16.0–41.0)	30.4 ± 9.5 (18.0–49.0)	
Time 400MWT [s]		485 ± 107 (288–771)**	694 ± 114 (477–879)**	
Stops 400MWT [number]		0.1 ± 0.3 (0.0–1.0)**	4.7 ± 3.3 (2.0–13.0)**	
Average daily steps [number]		6437 ± 3285 (2071–16643)*	4365 ± 1933 (2323–8732)*	
Average daily cadence [steps/min]		72.2 ± 7.6 (50.0–89.2)	68.1 ± 6.5 (58.0–78.4)	

*SD* standard deviation, *400MWT* 400-m walk test, *CIRS* cumulative illness rating scale, *FES-I* falls efficacy scale – international version, *SPPB* short physical performance battery

Statistically significant difference; *p* < 0.05*, *p* < 0.001**

No significant intervention effect in the number of daily steps (779 steps, 95%-CI[-561.5, -2119.3], *t*(89) = 1.16, *p* = 0.251), walking cadence (-0.1, 95%-CI[-3.5, 3.3], *t*(89) = -0.07, *p* = 0.945), time (34 s, 95%-CI[-24, 93], *t*(81) = 1.15, *p* = 0.253) and stops in the 400MWT (0.2, 95%-CI[-0.8, 1.2], *t*(82) = 0.37, *p* = 0.710) was found between the study groups at LO.

More stops were associated with poorer performance time in 400MWT after adjusting for sex, age, average daily steps and cadence at FO and additionally for group at LO. This effect was significant at FO (*B* = 0.013, 95%-CI[0.009, 0.026], *p* < 0,001) and LO (*B* = 0.014, 95%-CI[0.008, 0.073], *p* < 0,001). However, there was no significant association of average number of daily steps and walking cadence on stops in 400MWT.

**Fig. 2:**
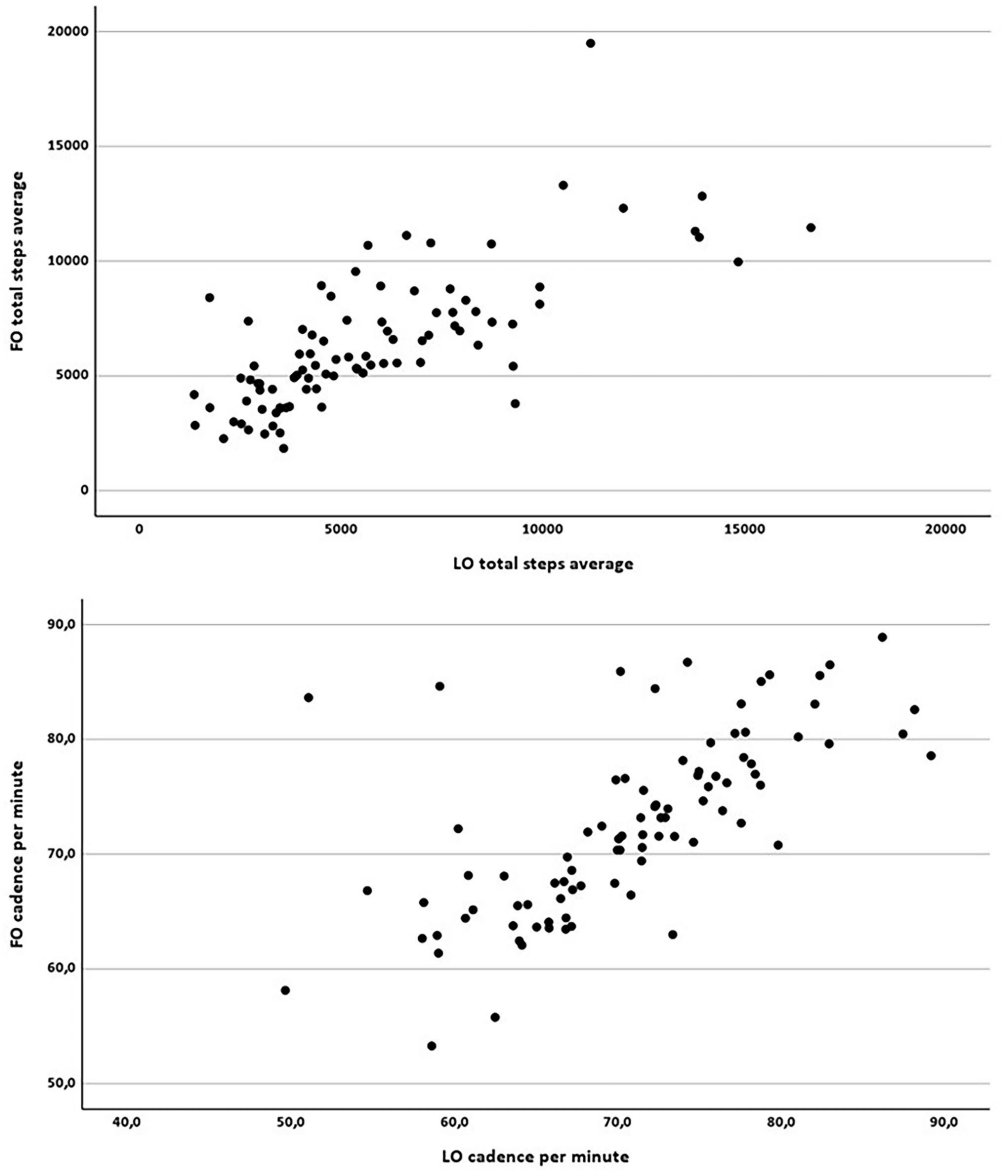
Change in average daily number of steps and walking cadence from FO to LO. The circles show the individual participants' steps (above) and cadence (below)

Average number of daily steps showed a significant decline at LO (*mdn* = 4861.00) compared to FO (*mdn* = 5577.00; asymptotic Wilcoxon test: *z* = -2.895, *p* = 0.004, *n* = 89). A paired t-test found a significant decline in walking cadence over time (*t*(88) = 2.589, *p* = 0.011) as well (Fig. 2).

In stepwise regression, in the final model, sex and time in 400MWT were identified as significant variables, while age, SPPB, FES-I, CIRS and handgrip strength were removed. After adjusting, time in 400MWT (*B* = -6.401, 95%-CI[-9.991,-3.3], *p* < 0,001) and sex (*B* = 1324.134, 95%-CI[281.953, 2397.470], *p* = 0.014) had a significant effect, indicating that a faster time in 400MWT is associated with on average more daily steps and that women tend to take on average more daily steps than men. At LO, significant effects in time (*B* = -9.616, 95%-CI[-14.828,-5.072], *p* = 0.002) and sex (*B* = 2397.237, 95%-CI[1346.685, 3601.390], *p* < 0.001), were found as well.

Regarding the average daily walking cadence, we again kept only the most relevant variables to avoid overfitting and therefore age, SPPB, FES-I, CIRS and handgrip strength were removed, while time in 400MWT was identified as significant variable. We also added height as covariate to control for its influence on walking cadence of the participating individuals. At FO, time in 400MWT (*B* = -0.018, 95%-CI[-0.028, -0.009], *p* < 0.001) was significantly associated with walking cadence, indicating that a faster time in 400MWT is associated with on average higher daily walking cadence. At LO, group was added as covariate and again a significant effect of time in 400MWT (*B* = -0.030, 95%-CI[-0.043, -0.016], *p* < 0.001) identified, which suggested that a faster time in 400MWT is associated with a higher daily walking cadence.

## Discussion

This sub-study evaluated the possible association of time and stops made in 400MWT and daily PA, assessed as average daily steps and walking cadence, of frail and sarcopenic older adults. This tool would then be able to provide individualized PA recommendations for the population. As expected, we observed that non-stoppers needed significantly less time in the 400MWT than multi-stoppers and that time was associated with stops made. Contrary to our hypothesis, stops did not show a significant association with average daily steps and walking cadence. Time, on the other hand, was significantly associated with average daily steps and walking cadence at both FO and LO. There was a significant decline in older adults’ gait parameters over time that is likely age-related.

The reasons for the demonstrated association between stops and time in 400MWT have been analyzed in one study so far [18], that attributed more stops to physical impairment or lower self-efficacy in walking ability. In other trials, the 6MWT test was examined in patients with COPD [15, 17] or PAD [16, 34] and more stops were attributed to leg pain or cardiovascular insufficiency. In our sub-study, reasons for stops or precise stopping duration were not assessed. Physical function (assessed with SPPB) showed no effects at any time point, but at FO, FES-I score was significantly different between non-stoppers and multi-stoppers. These results recommend further research into concerns about falling associated with stopping in the 400MWT. In addition, at LO but not at FO, multi-stoppers had a higher CIRS score, ergo disease status, which in turn was often the reason for more stops in the aforementioned studies. The fact that this difference in CIRS between multi-stoppers and non-stoppers only occurs at LO could be due to the participants’ increasing age within the study period.

Furthermore, the phenomenon of stopping while walking could be seen as a part of an individual’s walking strategy, with research showing that intermittent walking can reduce oxidative stress and improve cardiac autonomic function in older adults with hypertension [35] or lead to further distances walked and less fatigue in middle-aged participants with multiple sclerosis [19]. Older adults may therefore choose to stop so that they can continue to move or walk more calmly despite having enough reserves of energy left to complete a specific distance or task. Walking strategy can also differ in terms of the expected distance to be covered, as older adults performed longer walkways with higher stride velocity [20]. These studies could provide approaches to explain stopping in walk tests, although further research is needed to make more precise statements about the number of stops under the conditions of the 400MWT.

The most significant difference between non-stoppers and multi-stoppers seems to be the time in the 400MWT, as has also been stated by Vestergaard et al. (2009), with multi-stoppers in this sub-study having a slower performance compared to non-stoppers of 3.2 min at FO and 3.5 min at LO. These differences clearly exceed the minimally significant change estimates of the 400MWT (20–30 s) and also the substantial changes (50–60 s) [36], which supports the high clinical relevance of our results. Whether total time spent stopping influenced the association between stopping and mobility disability could not be shown [18]. Since time in 400MWT is a contributing factor to mortality prognosis [37, 38] and indicates mobility loss and subsequently mobility disability [23, 24, 39], it is a well-established parameter to evaluate physical function and mobility status in frail and sarcopenic older adults [3]. As it could be shown that more stops lead to a slower time, it is assumed that the number of stops might have an impact on the factors mentioned above as well and should be looked at in more detail in bigger samples.

The hypothesis, that stops in 400MWT are significantly associated with average daily steps and walking cadence, was not confirmed. To the best of the authors’ knowledge, this association has not yet been investigated in the 400MWT, but results from a trial investigating stops during 6MWT and 7-day step count showed that participants, that had to stop at least once during two 6MWTs had significantly lower 7-day step count, but not total SPPB score [16]. In our sub-study, the same results were found for SPPB score while difference in daily steps between multi-stoppers and non-stoppers has only been found at LO. This could possibly be due to the decline in various health factors. The multi-stoppers showed a significantly higher score in the CIRS at LO, which could have led to fewer steps. Additionally, SPPB score was lower and FES-I score was higher in multi-stoppers, yet this did not reach a significant level. Additionally, SPPB score was lower and FES-I score was higher in multi-stoppers, yet this did not reach a significant level. At this point, it should be mentioned that the SPPB generally increased during the intervention period. This might be due to the activating effect of the participation in the study or a regression to mean bias since a low SPPB was a central inclusion criterion at FO.

The multiple benefits of walking make it an easily accessible and healthy daily PA [40] and previous research stated, that among adults aged 60 years or older, an average of 6000 to 8000 steps per day even leads to decreased mortality risk [41]. However, in this sub-study, the multi-stoppers at FO and LO walked less than 6000 steps per day and thus might have a higher mortality risk than non-stoppers, something that would be interesting to follow-up on. Therefore, stopping in the 400MWT might be an early sign of a future decrease of health and everyday mobility. In older adults with knee osteoarthritis, it was shown that a lower time required in the 400MWT is predictive of a daily step count of ≥ 6000 steps [42]. Therefore, in this population, the rehabilitation program could be adapted and individual functional limitations addressed. Precise statements concerning the 400MWT in sarcopenic and frail older adults have not yet been made due to a lack of research. Nevertheless, Master et al. (2018) provided first insights into the possible application of the 400MWT as an analytical tool and then regulator of activity recommendations. A higher daily walking cadence is associated with better functional walking capacity in older adults [22, 43]. Part of this is gait speed, an important indicator for good health, that is inversely associated with mortality at all ages in both sexes [44]. However, the focus often lies on peak cadence and the daily time spent in it. The aim in most studies was, therefore, to adapt everyday activity by longer walking in medium-to-brisk cadence, which in turn could improve functional capacity. It must be taken into account that the sub-study, however, looked at the average daily cadence per minute and the above-mentioned activity recommendation approach may not be transferable to the sample. Ways to increase the average walking cadence or to maintain it over longer time intervals during the day would be a first approach to adapt activity patterns in this sample.

We also found significant associations of sex with daily steps, showing that women tend to walk significantly more daily average steps at both time points. These results may differ depending on the target group, for example, older adults take more steps if they are community-dwelling and able to be active outside their home [21]. Women also tend to complete more time in lower intensity PA and show less sedentary activity while men are more active for a shorter time in higher intensity [45]. After adjusting for height, sex did not show any significant association with average daily walking cadence. At FO, contrary to previous research normative values [46], cadence in women was lower than in men. This could be due to the significant age difference between the two sexes. There is a lack of clear statements about the different aging processes of men and women and the associated gender differences. The inclusion of body height in the analysis of parameters such as walking cadence seems generally recommendable, but values such as leg length could be more appropriate [9]. Other research showed an independence of cadence from body size and reported the adaptation of the female gait, especially speed, to the male gait, resulting in shorter steps and therefore, a higher cadence—probably for socio-cultural reasons [10, 47]. At LO, walking cadence in women was higher than in men. This shift in cadence goes hand in hand with the significantly higher number of average steps taken by women in everyday life. In our sub-study, SPPB and CIRS did not differ significantly between men and women. The higher number of daily steps in women can therefore be due to many factors, such as time spent doing household chores, living alone or better self-rated health [48]. More research is needed, to better understand the background of different activity patterns of frail and sarcopenic older men and women. Factors such as living situation and the individual character’s approach to everyday tasks and challenges should be taken into account to a greater extent.

Some limitations of the current sub-study have to be mentioned. We did not classify the stops into none and one or more stops [18], since we assumed a single stop could also occur by chance due to questions or comments from the participants during the test, leading to the “stops walking when talking” phenomenon [49]. Nevertheless, the distribution of non-stoppers (≤ 1 stop) and multi-stoppers (> 1 stop) was uneven (FO, non-stoppers: *n* = 84, multi-stoppers: *n* = 20; LO, non-stoppers: *n* = 69, multi-stoppers: *n* = 15) and general sample size in LO was diminished (n = 84) due to missing follow-up in some of the assessment tools, including 400MWT. Furthermore, the precise reasons for stopping were not recorded in this sub-study, as well as the individual and total time of the stops. A bigger sample size could include a higher number of multi-stoppers and provide more information on stops as indicator for mobility disability. It also has to be considered that some individuals might have over-performed in the 400MWT due to not walking at their usual gait speed as demanded in the protocol [14]. Previous research showed that performance in measurements of usual walking is influenced by aspects such as distance walked, instructions given and knowledge of being measured [50, 51]. In the sub-study, those lab-based effects were controlled by investigating the association of 400MWT with field-based daily PA. Additionally, half of the participants received an exercise intervention to improve their mobility with the 400MWT as primary outcome. This might have influenced the association between physical performance and daily activity. However, no significant differences in daily number of steps, walking cadence, time and stops in the 400MWT were present between the study groups so that the intervention effect seems to be neglectable for the analyses in this sub-study.

The sub-study has multiple strengths. First of all, to the best of the authors’ knowledge, a transfer of the performance in the 400MWT to daily PA in this population has not yet been investigated. The inclusion of stops during the 400MWT in analyses was also previously only performed in one study [18]. The study population of the sub-study consisted of a highly vulnerable group of frail and sarcopenic older adults, who were characterized in detail by numerous assessments. In addition, the participants were included for up to 36 months, which was previously only done in the LIFE collective [23]. The key assessment, the 400MWT, is a reliable, validated and widely used tool in frail and sarcopenic older adults [3, 14]. The study intervention was feasible, safe and effective, as the published results of the SPRINTT study showed significant effects on the reduction in the incidence of mobility disability [24]. In this sub-study, despite the smaller study sample, a significant association of time in 400MWT and average daily steps and cadence was found at FO and LO, suggesting a reliable relationship between the walk test and daily PA of frail and sarcopenic older adults.

In conclusion, non-stoppers needed significantly less time in 400MWT than multi-stoppers. However, stops were not significantly associated with average daily number of steps and walking cadence. Since time and stops were strongly associated, with the former predicting mortality and mobility disability, it is essential to further investigate the causes and duration of stops in the 400MWT. This parameter could provide additional information on walking strategies, daily PA and thus general health status of frail and sarcopenic older adults.

Age-related decline over time of daily average number of steps and walking cadence could be shown. In line with these age-related changes, a significant difference in the average daily number of steps between non-stoppers and multi-stoppers at LO was found. Additionally, time was significantly associated with average daily steps and walking cadence at both time points. As a widely used mobility assessment tool, the 400MWT would thus enable individual activity recommendations in frail and sarcopenic older adults in clinical settings or research. Importantly, wearable devices such as accelerometers can provide motivating and objective feedback on daily step count and cadence, supporting behavior change and promoting physical activity. Clinicians may use time in 400MWT to set personalized activity goals and track functional changes. Wearable devices can complement this by providing objective feedback on daily steps and cadence, supporting early intervention and improved outcomes.

## Data Availability

The datasets generated during this study are not publicly available, but are available from the corresponding author on reasonable request.

## References

[1] Alowaydhah S, Weerasekara I, Walmsley S, Marquez J (2024) Physical exercise for healthy older adults and those with frailty: what exercise is best and is there a difference? A systematic review and meta-analyses. Curr Gerontol Geriatr Res 2024:5639004. 10.1155/2024/5639004 39376725 10.1155/2024/5639004 PMC11458270

[2] Billot M, Calvani R, Urtamo A, Sánchez-Sánchez JL, Ciccolari-Micaldi C, Chang M et al (2020) Preserving mobility in older adults with physical frailty and sarcopenia: opportunities, challenges, and recommendations for physical activity interventions. Clin Interv Aging 15:1675–1690. 10.2147/CIA.S253535 32982201 10.2147/CIA.S253535 PMC7508031

[3] Cruz-Jentoft AJ, Bahat G, Bauer J, Boirie Y, Bruyère O, Cederholm T et al (2019) Sarcopenia: revised European consensus on definition and diagnosis. Age Ageing 48:601–601. 10.1093/ageing/afz046 31081853 10.1093/ageing/afz046 PMC6593317

[4] Rosenberg IH (1997) Sarcopenia: origins and clinical relevance. J Nutr 127:990S-991S. 10.1093/jn/127.5.990S 9164280 10.1093/jn/127.5.990S

[5] Fried LP, Tangen CM, Walston J, Newman AB, Hirsch C, Gottdiener J et al (2001) Frailty in older adults: evidence for a phenotype. J Gerontol A Biol Sci Med Sci 56:M146–M157. 10.1093/gerona/56.3.M146 11253156 10.1093/gerona/56.3.m146

[6] Cesari M, Marzetti E, Calvani R, Vellas B, Bernabei R, Bordes P et al (2017) The need of operational paradigms for frailty in older persons: the SPRINTT project. Aging Clin Exp Res 29:3–10. 10.1007/s40520-016-0712-5 28155179 10.1007/s40520-016-0712-5

[7] Xu J, Wan CS, Ktoris K, Reijnierse EM, Maier AB (2022) Sarcopenia Is associated with mortality in adults: a systematic review and meta-analysis. Gerontology 68:361–376. 10.1159/000517099 34315158 10.1159/000517099

[8] Clegg A, Young J, Iliffe S, Rikkert MO, Rockwood K (2013) Frailty in elderly people. The Lancet 381:752–762. 10.1016/S0140-6736(12)62167-9 10.1016/S0140-6736(12)62167-9 PMC4098658 23395245

[9] Frimenko R, Goodyear C, Bruening D (2015) Interactions of sex and aging on spatiotemporal metrics in non-pathological gait: a descriptive meta-analysis. Physiotherapy 101:266–272. 10.1016/j.physio.2015.01.003 25702092 10.1016/j.physio.2015.01.003

[10] Ko S, Tolea MI, Hausdorff JM, Ferrucci L (2011) Sex-specific differences in gait patterns of healthy older adults: results from the baltimore longitudinal study of aging. J Biomech 44:1974–1979. 10.1016/j.jbiomech.2011.05.005 21601861 10.1016/j.jbiomech.2011.05.005 PMC3124580

[11] Landi F, Cesari M, Calvani R, Cherubini A, Di Bari M, Bejuit R et al (2017) The “Sarcopenia and Physical fRailty IN older people: multi-componenT Treatment strategies” (SPRINTT) randomized controlled trial: design and methods. Aging Clin Exp Res 29:89–100. 10.1007/s40520-016-0715-2 28144914 10.1007/s40520-016-0715-2

[12] Theou O, Stathokostas L, Roland KP, Jakobi JM, Patterson C, Vandervoort AA et al (2011) The effectiveness of exercise interventions for the management of frailty: a systematic review. J Aging Res 2011:1–19. 10.4061/2011/569194 10.4061/2011/569194 PMC3092602 21584244

[13] Simonsick EM, Montgomery PS, Newman AB, Bauer DC, Harris T (2001) Measuring fitness in healthy older adults: the health ABC long distance corridor walk. J Am Geriatr Soc 49:1544–1548. 10.1046/j.1532-5415.2001.4911247.x 11890597 10.1046/j.1532-5415.2001.4911247.x

[14] Rolland YM, Cesari M, Miller ME, Penninx BW, Atkinson HH, Pahor M (2004) Reliability of the 400-M usual-pace walk test as an assessment of mobility limitation in older adults. J Am Geriatr Soc 52:972–976. 10.1111/j.1532-5415.2004.52267.x 15161464 10.1111/j.1532-5415.2004.52267.x

[15] Fernández-Sánchez MJ, García M, Correa-Ríos X, Cañas A, Lasso JI, Lutz JR et al (2015) Stops during the six-minute walk test and their correlation with new measurements of the test in patients with obstructive pulmonary disease. Rev Colomb Neumol 27:170–179

[16] Golledge J, Yip L, Fernando ME, Pinchbeck J, Rowbotham S, Jenkins J et al (2021) Relationship between requirement to stop during a six-minute walk test and health-related quality of life, physical activity and physical performance amongst people with intermittent claudication. Ann Vasc Surg 76:363–369. 10.1016/j.avsg.2021.03.038 33905859 10.1016/j.avsg.2021.03.038

[17] Andrianopoulos V, Wouters EFM, Pinto-Plata VM, Vanfleteren LEGW, Bakke PS, Franssen FME et al (2015) Prognostic value of variables derived from the six-minute walk test in patients with COPD: Results from the ECLIPSE study. Respir Med 109:1138–1146. 10.1016/j.rmed.2015.06.013 26143282 10.1016/j.rmed.2015.06.013

[18] Vestergaard S, Patel KV, Walkup MP, Pahor M, Marsh AP, Espeland MA et al (2009) Stopping to rest during a 400-meter walk and incident mobility disability in older persons with functional limitations. J Am Geriatr Soc 57:260–265. 10.1111/j.1532-5415.2008.02097.x 19170785 10.1111/j.1532-5415.2008.02097.x PMC2640434

[19] Karpatkin H, Cohen ET, Rzetelny A, Parrott JS, Breismeister B, Hartman R et al (2015) Effects of intermittent versus continuous walking on distance walked and fatigue in persons with multiple sclerosis: a randomized crossover trial. J Neurol Phys Ther 39:172–178. 10.1097/NPT.0000000000000091 26050076 10.1097/NPT.0000000000000091

[20] Najafi B, Helbostad JL, Moe-Nilssen R, Zijlstra W, Aminian K (2009) Does walking strategy in older people change as a function of walking distance? Gait Posture 29:261–266. 10.1016/j.gaitpost.2008.09.002 18952435 10.1016/j.gaitpost.2008.09.002

[21] Harris TJ, Owen CG, Victor CR, Adams R, Cook DG (2009) What factors are associated with physical activity in older people, assessed objectively by accelerometry? Br J Sports Med 43:442–450. 10.1136/bjsm.2008.048033 18487253 10.1136/bjsm.2008.048033

[22] Hoffman RM, Davis-Wilson HC, Hanlon S, Swink LA, Kline PW, Juarez-Colunga E et al (2024) Maximal daily stepping cadence partially explains functional capacity of individuals with end-stage knee osteoarthritis. PM&R 16:532–542. 10.1002/pmrj.13082 37819260 10.1002/pmrj.13082 PMC11006829

[23] Pahor M, Guralnik JM, Ambrosius WT, Blair S, Bonds DE, Church TS et al (2014) Effect of structured physical activity on prevention of major mobility disability in older adults: the LIFE study randomized clinical trial. JAMA 311:2387. 10.1001/jama.2014.5616 24866862 10.1001/jama.2014.5616 PMC4266388

[24] Bernabei R, Landi F, Calvani R, Cesari M, Del Signore S, Anker SD et al (2022) Multicomponent intervention to prevent mobility disability in frail older adults: randomised controlled trial (SPRINTT project). BMJ 377:e068788 35545258 10.1136/bmj-2021-068788 PMC9092831

[25] Guralnik JM, Simonsick EM, Ferrucci L, Glynn RJ, Berkman LF, Blazer DG et al (1994) A short physical performance battery assessing lower extremity function: association with self-reported disability and prediction of mortality and nursing home admission. J Gerontol 49:M85-94. 10.1093/geronj/49.2.M85 8126356 10.1093/geronj/49.2.m85

[26] McLean RR, Shardell MD, Alley DE, Cawthon PM, Fragala MS, Harris TB et al (2014) Criteria for clinically relevant weakness and low lean mass and their longitudinal association with incident mobility impairment and mortality: the foundation for the national institutes of health (FNIH) sarcopenia project. J Gerontol Ser A 69:576–583. 10.1093/gerona/glu012 10.1093/gerona/glu012 PMC3991140 24737560

[27] Folstein MF, Folstein SE, McHugh PR (1975) Mini-mental state. J Psychiatr Res 12:189–198. 10.1016/0022-3956(75)90026-6 1202204 10.1016/0022-3956(75)90026-6

[28] Fielding RA, Rejeski WJ, Blair S, Church T, Espeland MA, Gill TM et al (2011) The lifestyle interventions and independence for elders study: design and methods. J Gerontol A Biol Sci Med Sci 66A:1226–1237. 10.1093/gerona/glr123 10.1093/gerona/glr123 PMC3193523 21825283

[29] Gabriel KKP, Rankin RL, Lee C, Charlton ME, Swan PD, Ainsworth BE (2010) Test-retest reliability and validity of the 400-meter walk test in healthy. Middle-Aged Women J Phys Act Health 7:649–657. 10.1123/jpah.7.5.649 20864761 10.1123/jpah.7.5.649

[30] Bourke AK, Ihlen EAF, Helbostad JL (2019) Validation of the activPAL3 in free-living and laboratory scenarios for the measurement of physical activity, stepping, and transitions in older adults. J Meas Phys Behav 2:58–65. 10.1123/jmpb.2018-0056

[31] Tudorlocke C, Burkett L, Reis J, Ainsworth B, Macera C, Wilson D (2005) How many days of pedometer monitoring predict weekly physical activity in adults? Prev Med 40:293–298. 10.1016/j.ypmed.2004.06.003 15533542 10.1016/j.ypmed.2004.06.003

[32] Yardley L, Beyer N, Hauer K, Kempen G, Piot-Ziegler C, Todd C (2005) Development and initial validation of the falls efficacy scale-international (FES-I). Age Ageing 34:614–619. 10.1093/ageing/afi196 16267188 10.1093/ageing/afi196

[33] Miller MD, Paradis CF, Houck PR, Mazumdar S, Stack JA, Rifai AH et al (1992) Rating chronic medical illness burden in geropsychiatric practice and research: application of the cumulative illness rating scale. Psychiatr Res 41:237–248. 10.1016/0165-1781(92)90005-N 10.1016/0165-1781(92)90005-n 1594710

[34] McGrae McDermott M, Greenland P, Liu K, Guralnik JM, Criqui MH, Dolan NC et al (2001) Leg symptoms in peripheral arterial disease: associated clinical characteristics and functional impairment. JAMA 286:1599. 10.1001/jama.286.13.1599 11585483 10.1001/jama.286.13.1599

[35] Prasertsri P, Phoemsapthawee J, Kuamsub S, Poolpol K, Boonla O (2022) Effects of long-term regular continuous and intermittent walking on oxidative stress, metabolic profile, heart rate variability, and blood pressure in older adults with hypertension. J Environ Public Health 2022:5942947. 10.1155/2022/5942947 35140794 10.1155/2022/5942947 PMC8820939

[36] Kwon S, Perera S, Pahor M, Katula JA, King AC, Groessl EJ et al (2009) What is a meaningful change in physical performance? Findings from a clinical trial in older adults (the LIFE-P study). J Nutr Health Aging 13:538–544. 10.1007/s12603-009-0104-z 19536422 10.1007/s12603-009-0104-z PMC3100159

[37] Newman AB, Simonsick EM, Naydeck BL, Boudreau RM, Kritchevsky SB, Nevitt MC et al (2006) Association of long-distance corridor walk performance with mortality, cardiovascular disease, mobility limitation, and disability. JAMA 295:2018. 10.1001/jama.295.17.2018 16670410 10.1001/jama.295.17.2018

[38] Vestergaard S, Patel KV, Bandinelli S, Ferrucci L, Guralnik JM (2009) Characteristics of 400-meter walk test performance and subsequent mortality in older adults. Rejuvenation Res 12:177–184. 10.1089/rej.2009.0853 19594326 10.1089/rej.2009.0853 PMC2939839

[39] Chang M, Cohen-Mansfield J, Ferrucci L, Leveille S, Volpato S, De Rekeneire N et al (2004) Incidence of loss of ability to walk 400 meters in a functionally limited older population. J Am Geriatr Soc 52:2094–2098. 10.1111/j.1532-5415.2004.52570.x 15571549 10.1111/j.1532-5415.2004.52570.x

[40] Lee I-M, Buchner DM (2008) The importance of walking to public health. Med Sci Sports Exerc 40:S512–S518. 10.1249/MSS.0b013e31817c65d0 18562968 10.1249/MSS.0b013e31817c65d0

[41] Paluch AE, Bajpai S, Bassett DR, Carnethon MR, Ekelund U, Evenson KR et al (2022) Daily steps and all-cause mortality: a meta-analysis of 15 international cohorts. Lancet Public Health 7:e219–e228. 10.1016/S2468-2667(21)00302-9 35247352 10.1016/S2468-2667(21)00302-9 PMC9289978

[42] Master H, Thoma LM, Christiansen MB, Polakowski E, Schmitt LA, White DK (2018) Minimum performance on clinical tests of physical function to predict walking 6,000 steps/day in knee osteoarthritis: an observational study. Arthritis Care Res 70:1005–1011. 10.1002/acr.23448 10.1002/acr.23448 PMC5904009 29045051

[43] Gonzales JU, Shephard J, Dubey N (2015) Steps per day, daily peak stepping cadence, and walking performance in older adults. J Aging Phys Act 23:395–400. 10.1123/japa.2014-0049 25134524 10.1123/japa.2014-0049

[44] Studenski S (2011) Gait speed and survival in older adults. JAMA 305:50. 10.1001/jama.2010.1923 21205966 10.1001/jama.2010.1923 PMC3080184

[45] Hansen BH, Kolle E, Dyrstad SM, Holme I, Anderssen SA (2012) Accelerometer-determined physical activity in adults and older people. Med Sci Sports Exerc 44:266–272. 10.1249/MSS.0b013e31822cb354 21796052 10.1249/MSS.0b013e31822cb354

[46] Hollman JH, McDade EM, Petersen RC (2011) Normative spatiotemporal gait parameters in older adults. Gait Posture 34:111–118. 10.1016/j.gaitpost.2011.03.024 21531139 10.1016/j.gaitpost.2011.03.024 PMC3104090

[47] Bruening DA, Baird AR, Weaver KJ, Rasmussen AT (2020) Whole body kinematic sex differences persist across non-dimensional gait speeds. PLoS ONE 15:e0237449. 10.1371/journal.pone.0237449 32817696 10.1371/journal.pone.0237449 PMC7440644

[48] Stalling I, Gruber M, Bammann K (2024) Sex differences in physical functioning among older adults: cross-sectional results from the OUTDOOR ACTIVE study. BMC Public Health 24:1766. 10.1186/s12889-024-19218-x 38956507 10.1186/s12889-024-19218-x PMC11221023

[49] Lundin-Olsson L, Nyberg L, Gustafson Y (1997) Stops walking when talking as a predictor of falls in elderly people. Lancet 349:617 9057736 10.1016/S0140-6736(97)24009-2

[50] Krumpoch S, Lindemann U, Becker C, Sieber CC, Freiberger E (2021) Short distance analysis of the 400-meter walk test of mobility in community-dwelling older adults. Gait Posture 88:60–65. 10.1016/j.gaitpost.2021.05.002 34000486 10.1016/j.gaitpost.2021.05.002

[51] Middleton A, Fritz SL, Lusardi M (2015) Walking speed: the functional vital sign. J Aging Phys Act 23:314–322. 10.1123/japa.2013-0236 24812254 10.1123/japa.2013-0236 PMC4254896

