# Correction: Association between the 400-m walk test and sensor-based daily physical activity in frail and sarcopenic older adults

**DOI:** 10.1007/s41999-026-01410-4

**Published:** 2026-01-26

**Authors:** Jana Rogler, Sebastian Krumpoch, Ellen Freiberger, Ulrich Lindemann, Robert Kob

**Affiliations:** 1 Department of Internal Medicine-Geriatrics, Institute for Biomedicine of Ageing (IBA), Friedrich-Alexander-Universität Erlangen-Nürnberg, Nuremberg, Germany; 2 Department of Geriatrics, Robert Bosch Hospital, Stuttgart, Germany

**Correction: European Geriatric Medicine (2025) 16:1799–1810** 10.1007/s41999-025-01262-4

In Fig. 2 the same scatter plot was shown for the total average daily steps and the cadence per minute.
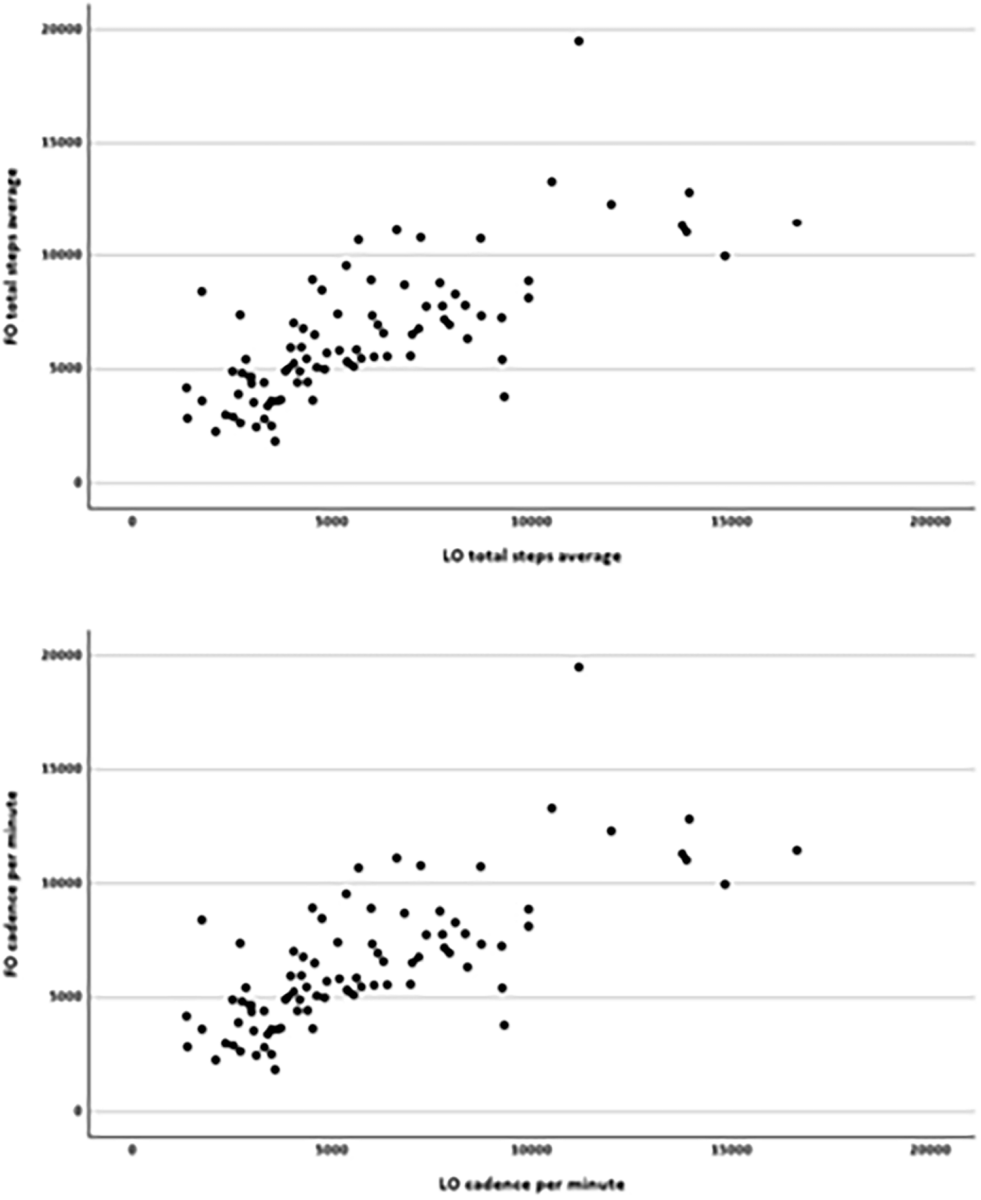


The correct and complete figure is now presented here.
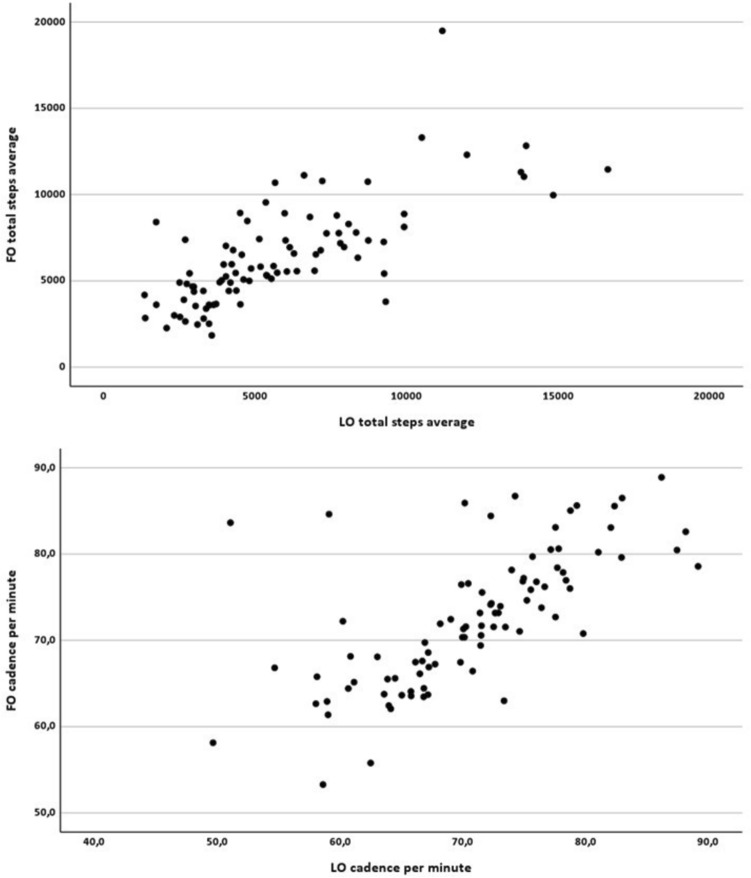


The original article has been corrected. Rogler et al. apologize for this error.

